# Proceedings of the Twentieth Annual Meeting of the American Dental Association

**Published:** 1880-09

**Authors:** 


### PROCEEDINGS OF THE TWENTIETH ANNUAL MEETING OF THE AMERICAN DENTAL ASSOCIATION.

The regular annual meeting of this body was held August 3–6, 1880, in Huntington Hall, in the Institute of Technology, Boston, Mass.

At 11 o'clock the Association was called to order by President L. D. Shepard, of Boston, and prayer was offered by the Rev. Dr. Studley, of Newton. The roll of qualified members was called, and about a hundred responded. President Shepard then made a brief informal address, welcoming the members of the Association to Boston. The only meeting of the preceding nineteen annual sessions held in Boston was in 1866.

Dr. Fillebrown, of Portland, Me., from the local Committee of Arrangements, stated the hours of daily session to be from 10 A. M. to 2 P. M., and from 7½ P. M. till adjournment. He also stated the regulation that there be no smoking in the building, and said, further, that, as the Association was purely scientific, it was expected that no one would make the meetings a place of merchandise. The report of the committee was accepted.

Proposed constitutional amendments were then taken up. The first, which was presented in writing at the last session, was to the effect that all papers to be published in the transactions of the Association shall be in the hands of the Publication Committee before September 1st, and in such shape that they will require no material alteration afterwards, and that all papers read before the Association, and all cuts used for illustration, are the exclusive property of the Association, and that they shall be published for the exclusive benefit of the Association. Attention was called by Dr. Buckingham to the undesirability of the regulation that the papers presented shall be published in no other place than in the official volume of the proceedings. Dr. Thomas, of Detroit, moved an amendment that dental journals be authorized to publish the papers. The Chair stated that copying from the official publication was not prohibited by the amendment, which simply required that the Association should have the first publication of the papers. In previous years, papers were in type at the time they were read, and were published by the authors in September dental magazines before they could be published by the Association. It was this practice which the amendment was designed to prevent. In view of this explanation, and the statement that it was not designed to prevent the daily press from making reports, the amendment was withdrawn. The original amendment was made to conform to this explanation, by striking out "exclusive" before "benefit," by a standing vote of 45 yeas to 5 nays. The amendment was then adopted.

The second amendment made limited the Committee on Nominations and Volunteer Essays to Volunteer Essays alone, and was so adopted. The Publication Committee was appointed from the whole Association as well as from the Executive Committee. Members suspended, as well as expelled, from their local society were declared to have forfeited their membership in the Association. All these amendments were presented in writing at the last annual meeting, and were adopted.

The Committee on Credentials then reported ninety-nine delegates present. Dr. Cushing next read a letter from Dr. Birge, of San Francisco, who resigned two years ago, asking that the resignation be expunged, and that he be reinstated upon the payment of his back dues—$15. Upon motion of Dr. Cushing, the doctor was reinstated upon his own terms. His letter was referred, upon motion of Dr. George F. Waters, to the Publication Committee, with power to print it. Dr. Marvin raised the point whether the vote of the Association was not irregular, inasmuch as Dr. Birge's connection with the Association had wholly ceased, and that he must come in by the regular way prescribed by the constitution for new members. The Chair considered Dr. Marvin's point well taken, and that there was no provision in the constitution for the reinstatement of a member who voluntarily resigned. Dr. Wetherbee, expressing the utmost good feeling for Dr. Birge, moved that the vote be rescinded. Dr. McKillops raised the point that the whole proceeding was out of order, and the Chair sustained the point. Dr. Cushing then moved that the vote passed at the resignation of Dr. Birge, two years ago, be reconsidered. The point of order was made by Dr. Alport, of Chicago, that the motion was out of order, as such a motion could be made only after one year. This point was sustained. Dr. Bogue moved that the Secretary answer Dr. Birge's letter, by saying that his request could not be legally granted, but that the Association would be pleased to have him become a member by the regular channel. This motion was carried unanimously. Dr. Alport, by common consent, spoke of the importance of the provision that expelled or suspended members of local societies, lose membership in the Association, objecting to it as too stringent. Dr. Priest offered a resolution that members who have resigned may be reinstated by unanimous vote. This, being a constitutional amendment, went over to next year. Dr. Fillebrown moved, as the opinion of the Association, that unless a member has had a vote of expulsion or suspension passed upon him by his local society, the clause of the constitution referred to did not affect his membership in the Association. The fact of a man having voluntarily withdrawn, or his name having been dropped from his local society on account of nonpayment of dues or other kindred cause, should not exclude him from the Association. The resolution was ruled out of order, on a point taken by Dr. Morgan, on the ground that it sought to annul the constitution.

The Chair was sustained, Dr. Fillebrown's resolution was ruled out, and the Association proceeded to the next business.

Reports of the sections were in order, with section 2 first on the list; but, as it was not ready, reports from special committees were called for. It was stated that the report of the special Committee on the Union of the Societies would be first in order for the evening session. These societies are the American Dental Association, the American Dental Convention, and the Southern Dental Association. At 2 o'clock the Association adjourned to half-past 7 in the evening.

---

### EVENING SESSION.

The Association was called to order at 8 o'clock, by the President. The minutes of the morning session were read and approved.

Dr. R. F. Hunt, of Washington, D. C., sent in his resignation as member of the Association; it was accepted The report of the special committee on the consolidation of the three societies mentioned above, was next taken up. Its points were as follows: It would not be wise or expedient for the Association to disband in order to assist in the formation of a new organization. It would not aid in the advancement of the profession to form such new organization. The committee recommended that no action be taken. The report was accepted, and adopted unanimously without debate.

The courtesies of the floor were extended to Dr. George W. Field, of London, who was present.

The report of section 2, on Dental Education, was next in order, but it was not ready, and additional time was granted. Section 3, on Dental Literature and Nomenclature, was granted further time. The President of section 4, on Operative Dentistry, Dr. Keely, was absent, and no report was made from that section. Of section 5, only Dr. Barrett, of Buffalo, had prepared a report, but he was absent. Section 6 had held a meeting in the afternoon. It has two written reports and one report of cases. It had been voted in the afternoon that the author of each paper read his own production.

Dr. Morrison, who had the case to report, was the first speaker. The case was that of a patient who suffered from a sensitive tooth and swollen cheek, but who experienced relief and ultimate recovery after a discharge of matter, which was made through the nostril. Dr. Morrison believed that superficial treatment is common in similar circumstances, especially with young subjects, in cases of inflammation of the antrum.

Dr. C. A. Brackett then read his paper. His first point was the flow of saliva, which impedes operations in very wet mouths. Belladonna is given to prevent saliva from flowing, but it is a powerful narcotic. The peculiar action of belladonna has been well understood by physicians, but its properties have not been put into practical use by dentists. Dr. Brackett's second point was the necessity of care on the part of dentists not to injure their patients' teeth by the use of acids. He cited a case in which a good dentist injured a patient's teeth by the use of tannic acid. He had tested some of the agencies used by dentists. Among the substances which he found injurious were tannic acid, alum, (which destroys with great energy the elements of the enamel, is often used by dentists, and is often an ingredient of tooth-powders), phosphoric acid, dental obtundent (Dr. Wetherbee's), salycilic acid (which is found to induce white decay in the tooth), and ordinary alcoholic tincture of myrrh (the specimen shown had been kept some time, but every test made showed the acid reaction). Glycerine, absolute alcohol, aconite root, and compressed chlorate of potassa were the only substances which gave absolutely no acid reaction, but it would not follow that their compounds would be harmless. One part glycerine and two parts of aconite root combined showed acid reaction. Deliquescent cloride of zinc readily showed acid reaction ; also, Smith's oxychloride. Several cements and paste also gave clear proof of containing acid, but in combination with powders the acidity disappears. Enough had been proved to show the active acidity of medicaments used by dentists, and to prove the importance of great care on the part of the operator. Dr. Brackett's third and last point was in reference to the prevention of pain in the excavation of dental cavities. It is a recognized fact that dry cavities are less sensitive than wet ones. Hitherto mechanical means alone have been tried, but chemical means should be tried to produce dryness in the tooth. The best materials for this purpose are glycerine and absolute alcohol, in combination with tincture of aconite root or other non-acid substances. Dr. Shumway, who has used these materials, claims that his ideas are at the basis of the new invention, naboli, the credit of which is claimed also by Dr. C. G. Davis, of New Bedford. Dr. McDougal regarded heat, applied in the form of the hot-air blast, as the best obtundent known. He also claims to have discovered, independently, the value of the use of glycerine and absolute alcohol as obtundents. Dr. Brackett regarded the claims of these dentists as honestly made, each investigator having arrived at his result independently, by personal experiments. Dr. Brackett took up the subject of naboli, giving several analyses of it. The analysis of

James F. Babcock of the contents of the four bottles in which naboli is contained, showed the first to contain petrolium oil, with a little tannic acid; the second to be like the first, with a greater proportion of acid; the third to consist of glycerine perfumed, with tannic acid in still greater proportion; the fourth to consist of wood naptha, or methylic alcohol, a product of the distillation of wood, holding in solution various impurities, which give it its peculiar odor and acid reaction. Dr. Brackett did not express a high opinion of naboli, and counselled the dentists to be slow in paying high prices for licenses or in binding themselves by hard conditions.

Dr. Odell, of New York, read a very interesting paper upon "*Taking Cold*." He made quite clear the point that catarrhal affections are largely depending upon a general weakening of the system, together with a want of fresh air, sunlight, sleep and good food.

The Association now adjourned till 10 A. M. to-morrow.

---

SECOND DAY—MORNING SESSION.

At 10½ o'clock the Association was called to order, the President in the chair.

The minutes of the last session were read and approved.

Dr. C. S. Stockton, of Newark, N. J., presented the report of the Committee on National Directory. The recommendation was made that the lists of dentists be revised every year, and that the lists made by dealers in dentists' goods be not accepted as authoritative. Reports were presented from nearly all the States, giving a list of members of the Association, and a special report was made from New Jersey, showing a nearly perfect condition of the directory of that State. The report was accepted as a report of progress. Dr. H. J. McKellops, of St. Louis, offered the following resolution:

"WHEREAS, It is desirable that the dental profession, as a body, should give countenance to no other material for filling teeth but the best, and encourage the greatest attainable thoroughness in the treatment of diseased teeth; and

"WHEREAS, Of late years, many different alloys for the compounding of amalgams have been presented to the profession for their adoption, and put upon the market by the manufacturers, warranted to resist chemical change and thermal influence; and

"WHEREAS, Some few members of the profession have seen fit to engage in the manufacture of said alloys for the formation of amalgams, and have used their position as professors to promulgate the doctrine of the superiority of amalgam compounds over all material as a filling for the preservation of the teeth; and

"WHEREAS, Other members of the profession, holding responsible positions in some of our best colleges, have permitted their names to appear in connection with the said advertisements, setting forth the many fine qualities and rare virtues of these compounds, thereby giving countenance of a scientific character to the sale and use of these compounds; and

"WHEREAS, Many members of the profession are but slightly acquainted with the many principles involved in the choice of the best material for filling, and very naturally look up to the professors, in their scientific character, as authority in all such matters; and

"WHEREAS, There is a peculiar impropriety in professors lending their names to the advertisements of these or any other compounds, inasmuch as charlatans lose no opportunity to exhibit and parade the names of these professors before an ignorant community, in justification of their constant use of an inferior material for filling teeth; now, therefore, be it

"*Resolved*, That this Association discountenance, in a formal and emphatic manner, the pernicious practice of members of the profession, but more especially those holding high positions in our schools, of allowing their names to appear in advertisements as indorsing either any special material or compound for filling teeth, or any therapeutic agent whatsoever."

Dr. W. H. Atkinson, of New York, spoke against the resolution, taking the ground that the dentists could not know about all amalgams, and that it would be presumptuous to condemn them without investigation. If he meant Chase and Flagg, why not come out and say it. Dr. E. T. Darby, of Philadelphia, spoke for the resolution. Dr. H. W. Morgan, of Nashville, also favored it, thinking it was a question of ethics which was involved. Dr. W. W. Allport, of Chicago, also supported the resolution, and, after further discussion by other members, Dr. Shepard, who had taken the floor, offered an amendment, to be added at the end of the resolution, "the nature or composition of which they are ignorant." Objection was made, and the amendment was withdrawn. Dr. Buckingham contended that the dentists had a right to recommend materials which were known to be good. The resolution was adopted with a few dissenting voices.

A further report of the Committee on Credentials was made, showing quite an increase in the attendance.

A unanimous vote was passed requesting the proprietors of dental exhibitions to close their rooms during the sessions of the Association. Then the regular order of the day was taken up—the first subject being a discussion of Tuesday evening's report. Dr. Morgan spoke of his experience, and thought that necrosis of the antrum should be treated like necrosis in any other place. Dr. Salmon spoke briefly, and Dr. J. G. W. Werner, of Boston, did not think necrosis of the antrum should be treated like other necrosis. In a recent case of his, he used nothing to irritate the parts, but kept them well washed, clean and warm. He warned against irritation. Dr. Atkinson made a distinction in irritation. Only that which goes beyond nutrient irritation is injurious. Abscess of the antrum never comes till there is occlusion of the passage of the antrum. He had known abscess of the antrum to last for ten years. If the bones are diseased, there is only one remedy—a surgical operation. The system should be carefully built up till nature makes a clear demarcation between the living and dead bone. Dr. Atkinson held that there would be no inflammation without constitutional deterioration. Dr. Werner claimed that, in certain stages, warmth is required, and that if cold was applied in the treatment of diseased antrum, the dentist would regret it. Dr. Mills related a case which was in his care last winter. His treatment was the opposite of that advised by Dr. Werner. He applied injections, and succeeded in effecting a cure.

Section 6 was then passed, and section 4 taken up by special motion. Chairman Keely was absent, and Dr. Darby presented the report. Only one paper upon Operative Dentistry was presented, and that not by a member of the Association, but by a member of a local Western society, but it had been indorsed by the censors. Dr. Darby spoke of the replantation and transplantation of teeth as worthy of discussion by the Association. Discussion was also suggested upon the use of phosphates, and the preparation of gold. Dr. Joshua Tucker was invited to address the meeting. He was escorted to the platform by Dr. Allport. His remarks were upon the use of soft gold in filling teeth, explaining its advantages and the best method of inserting it. He stated he was strongly of the opinion that the old system of filling teeth with soft gold, with pressure against the walls of the cavity, was the best method, believing that it conduced most to the preservation of the teeth. The paper mentioned above, by John J. R. Patrick, of Belleville, Ill., was then read by Secretary Cushing It was upon "Oral Electricity and the New Departure." He said that the frequent failures in filling teeth with gold were too apt to lead the practitioner to seek for the cause of failure outside of himself, for no man who honestly does his utmost is willing to suspect his manipulative skill. He at once seeks for redress in a series of speculations, and as the result imagination unconsciously takes the place of reason. There are other causes which have been as fruitful of failure as defective manipulation. First, the continual appeals on the part of patients for inexpensive work; for it is frequently the case that a well-dressed man, wearing diamonds and jewelry, will request his dentist to use the cheapest possible material, and a majority of women will spend more money in one year with their milliners than they spend with dentists in ten years, and still feel the tax of the dentist the heavier of the two. Second, the ease to the patient and the operator in the use of plastic materials. Third, the more profitable employment, in a monetary point of view, to the operator. Fourth, the deep interest shown by the champions and promulgators of this old system revived of saving teeth in putting compounds of their own manufacture upon the market. He proceeded to discuss and criticise Dr. J. Foster Flagg's pamphlet on the basal principles of the "new departure," as well as the arguments of Drs. Palmer and Chase, the other two champions of the "new departure." Professor Flagg had said that teeth are safer in the hands of the majority of practitioners turned out from our schools, with a plastic material for filling, than with gold. The essayist thought so much the worse for the schools. If a dentist cannot fill teeth with gold, he ought not to be allowed to practice. He said that, in the selection of filling materials, to secure a good result it was indispensable that the material used should fit the walls of the cavity perfectly, and exclude completely all those external agencies which induce decay; that it should be solid and undivided in all its parts, fitting it to resist all pressure likely to be brought to bear against it, and, finally, that it should resist to the fullest extent chemical action, thermal influence and attrition.

So long as they were confined to the use of metals for filling material, there is no metal, or combination of metals or substances, that has yet been found that can take the place of gold for that purpose. In his conclusions, he held that the word battery was not applicable to any arrangement of elements present in the mouth. Messrs. Chase and Flagg, he said, were inconsistent with themselves, and their experiments generally worthless. A detailed review was made of Dr. Chase's writings, and their discrepancies pointed out, to the interest and amusement of the Association. The essayist denied the existence or possibility of electric currents in the mouth, except by having a tin filling opposite a silver filling, each affected by the moisture and acids of the mouth, when a feeble action would take place whenever the surfaces of the fillings were brought together. Dr. Palmer's views were also criticised freely. Silver and mercury were regarded as unsuited for the preservation of teeth, on account of their affinity for sulphur, which is found abundantly in the human body. Iodine is also readily disposed to unite with mercury. Dr. Patrick believed that no substance for filling had yet been found equal to gold, and the paper mentioned the important respects in which gold is superior to any other substance for filling teeth. He discussed amalgams, the contraction, conduction and expansion of metals. He recommended the disuse of all amalgams as filling material. His final warning was in the following words: "When an individual, either by bad counsel or a false economy, has been subjected to mercurial or amalgam treatment for diseased teeth, we find that fifty parts of the mercury out of the sixty used in forming the amalgam is vaporized by the heat of the body in a few years, and taken into the system in small but regular quantities, the most potent manner of administering mercury for constitutional effect. Should not this be sufficient to induce every conscientious practitioner to discard all amalgams from the list of filling material, and be the means of inducing others to be less presumptuous in their speculations, and more honorable and resolute in their practice."

Dr. Atkinson criticised the paper severely, as "wretched platitudes and miserable verbiage," and "wretched drivelling of misconceptions and misstatements," and "wretched drivelling idiocy of some wretch who sent his paper here." [Hisses.] "Well, hiss if you want to. Geese always hiss, and sometimes ganders." If the real flour in that mess of trash had been given alone, it would have taken about two minutes. Dr. Buckingham protested against the denunciation, and said there was sense in the paper worthy of the consideration of the Association. He believed that electricity does exist in the mouth, but electricity and the incompatibility of metals with tooth substance are difficult matters to discuss, and should be carefully investigated. No paper should be repudiated as this one had been. [Applause.) Dr. Atkinson said that he did not find fault with the study of the depths of the science, but he characterized the paper as he did, religiously. He has waited long for gray headed men to come to the conception that electricity is but a mode of motion. Only two compounds of mercury are deleterious to the body—simple chloride, which is calomel, and the bichloride, which is corrosive sublimate. Dr. Buckingham asserted that the action of poison on the system could not be explained.

Adjourned till evening.

SECOND DAY—EVENING SESSION.

The carriage drive that had been arranged for the afternoon was somewhat delayed by the rain, which prevented the assembling of the Association till 8½ o'clock, when the President, Dr. Shepard, called the body to order. The minutes of the morning session were read and approved.

The Auditing Committee reported that the books and papers of the Treasurer and Publication Committee had been examined, and found correct. This report was accepted.

Dr. Mills, of New York, moved that a committee of three be appointed to prepare resolutions upon the death of Dr. S. S. White, of Philadelphia.

Next in order was the discussion of papers and reports on Operative Dentistry. Dr. Allport opened the discussion. He had long been opposed to the exclusive use of cohesive gold in filling decayed teeth. It has its proper use, but that use is not filling to prevent decay. It should be used to give a polished surface or in restoring the form of the tooth; but those things were very different from filling a cavity. It is necessary to find a substance which will make a perfect union with the walls of the cavity. Cohesive gold is hard to manipulate, and does not easily adapt itself to the cavity. A substance may be hard, but be full of holes. This is the case with cohesive gold, and the microscope will reveal the holes. Forty years ago the ordinary operators saved more teeth with soft gold than they do now. Yes, more than the college graduates, save to-day. Nothing but the most extraordinary skill and the greatest care will save teeth with cohesive gold, but soft gold will preserve them. Decay goes on around the cohesive gold. At the end of five years there will be a fine filling and a blue, decayed tooth. Moisture will get in between the filling and the gold, which will inevitably cause decay. Dr. Morgan questioned Dr. Allport as to what he meant by a perfect filling; and he said he meant one which exactly filled the cavity. If he wanted to build up the form of the tooth, he would use cohesive gold; but for the simple purpose of filling a cavity, the softer he could get the gold the better. Cohesive gold requires several retaining pits to keep it in place, and is a most deceptive substance for inexperienced operators, who think they have got a fine operation when they have not. Dr. Morgan protested against the views of Dr. Allport, saying that he (Dr. A.) assumed, because he could not use cohesive gold, that nobody else could. It was simply his acknowledgment of his want of ability to do that thing. He (Dr. M.) could make, and has made, good fillings with cohesive gold. The ideal filling is one in which the cavity is so shaped as to retain the gold, in which no decay is left; in which the cavity is filled, and in which the gold is one solid mass when the operation is ended. Dr. Morgan asserted that Dr. Allport was mistaken as to his statement about fillings forty years ago. Dr. Fillebrown also took up the cause of the cohesive gold, asserting that it would perfectly fill cavities as well as soft gold, and that it could be used without a mallet, and with from three to six pounds pressure, instead of twenty to twenty-five, which is required in welding soft gold. This cohesive gold is the most plastic form of gold which can be used. He knew that fillings were made which did not turn blue for twelve or fifteen years. Dr. Thomas said that no two dentists fill teeth in the same way, and it is folly to protest against any man's practices in filling. He thought that when cohesive gold was first introduced dentists went wild over it, as they have over many things since. He thought that there was a middle ground as to the practice of filling with gold. The longer he was in business the less he was inclined to dogmatize as to which was the best way to fill teeth. [Applause.] In his practice he came across fillings made thirty years ago which were now preserving teeth as perfectly as ever. Yet they are made of gold so soft that the excavator can be pushed through it. He believed Dr. Allport made a good point as to the hardness of cohesive gold; it is very deceptive. Young operators should be taught to use soft gold before they use cohesive gold. They get to using larger and larger pellets, and the fillings become more and more porous. Cohesive gold, under pressure, will draw away from the walls of the cavity toward itself, at the centre. It will curve and crink and crack, and make a poor filling. Dr. J. Taft protested against special pleading. He believed that cohesive gold can be adapted to the walls of any cavity as perfectly and easily as non-cohesive gold. [Applause.] For every case of easily perforated soft foil filling which has been successful for many years, he could find a case of a cavity with no filling at all which has kept for an equal length of time. Operators who find that cohesive gold draws away from the walls of the cavity, either have very defective instruments, or they do not know how to use them. Any manipulator of ordinary ability, with proper instruments, can adapt accurately the gold to the walls of the cavity. Dr. Taft also defended the use of retaining pits and angles in cavities to be filled, though he would not have them weaken the structure of the tooth. It is very important to have the first piece of gold retain the exact position where it is first placed and not be removed and replaced. As to the force required to condense cohesive and non-cohesive gold, he thought that, with equal amounts under the instruments the number of pounds pressure in each case would be about the same. He has not found decay the rule, or anything like it, in regard to fillings of several years' standing.

Dr. Thomas spoke again, by common consent, to explain what he meant by the burring of cohesive gold. He believed the danger from that kind of gold was from the ends of the tubuli, and not from moisture from the outside, and cohesive gold does not fit exactly the walls of the cavity not exposed to the air.

Dr. Atkinson said that, in filling, no pulp should ever be exposed. He believed that young men should be taught by observing the dentists at work upon their best-loved patients, and see how it is done. Less depends upon a knowledge of method than upon a knowledge of principles in dentistry.

Adjourned.

---

### THIRD DAY—MORNING SESSION.

The Association was called to order at 9½ o'clock, the President, Dr. Shepard, in the chair. The minutes of the last session were read, corrected and approved.

On motion of Dr. Essing, a vote of thanks was extended, with great cordiality, to the dentists of Boston for the very interesting carriage drive about the city and suburbs of Boston given to the members of the Association on yesterday afternoon.

The President appointed Drs. Atkinson, of New York; Pierce, of Philadelphia; Taft, of Cincinnati; Morgan, of Nashville, and Mills, of Brooklyn, the committee to draft a resolution on the death of Dr. S. S. White.

Dr. Fredericks, of New Orleans, re-opened the discussion on the subject of the relative merits of cohesive and soft gold for filling. He was followed by Dr. Lord, of New York, and Dr. Stockton, of Newark, N. J. The last-named speaker also spoke of the fault found with the prices charged for their work. He thought dentists were partly to blame for this, and that there ought to be some kind of understanding among the profession on this question. They were intrusted with the knowledge, and consequently the duty, to preserve the teeth, and should conscientiously work upon the principal of moral responsibility. Further remarks were made upon the subject by Dr. Cook, of New York, Dr. Allport, of Chicago, Dr. Webb, of Lancaster, Pa., and Dr. Wetherbee, of Boston. Dr. Mills spoke of the importance of attending to the cleanliness of a patient's teeth, and the health of the mouth. It was an exception that a patient came into his hands in whose mouth there was any evidence of an effort to accomplish that object. He had been practicing in Brooklyn for four years, and only in one case had he found the marks of such an effort. He considered this fact deplorable, and believed it remained for the young members of the profession to come up to the standard on this point. With reference to the variety of opinions on the subject of the best materials and the best modes of operation in filling, he did not feel like denouncing an operator's system or practice because they were not the same as his own, or because similar means were not employed by both to obtain the same results; but the foundation plank in the successful filling of teeth was, in his opinion, particular regard for cleanliness.

Dr. G. F. Waters, of Boston, spoke of how much depended upon patients themselves as to the beneficial results of dental treatment and the preservation of the teeth. He illustrated the point by relating an incident in his practice. It was the case of a lad who came to him, many years ago, to have his teeth cleaned, and who accosted him on the street twenty-five years later, and exhibited the most beautiful set of teeth he had ever seen as the result of the perfect care the patient had given his mouth, to which he was actuated by a startling warning administered by the doctor when he cleaned the teeth.

Dr Talbot, of Chicago, exhibited and presented to the Association a new kind of instrument invented by him for excavating cavities.

Section No. 5, on anatomy, physiology, histology, microscopy and etiology, was next called upon to report. Dr. George H. Cushing, of Chicago, Chairman of the section, stated that the only paper they had to present was by Dr. Barrett, of Buffalo, N. Y., who then proceeded to read an essay on anæsthesia, which he defined to be "paralyzation of the nerve tissues." The essayist related the results of many interesting experiments made by him in connection with his study of anæsthetics, and discussed the effects produced upon the human system by the use of the various means which produce artificial anæsthesia. His opinion was that, if it be properly administered and closely watched, chloroform is not as dangerous as it has been represented, but that its administration absolutely demands the services of a skilled expert; for it is more powerful than any other agent to produce narcosis, and requires greater care in its exhibition. Especially should the eye be watched, for though in its later stages the pupil is expanded, care must be taken that the entire relaxation of the iris, as in coma, be not approached. In ether narcosis the excitement stage is seldom attended with such violence as in chloroform, and hence there is less danger at that time, for the cases in which ether has proved fatal through paralysis of the heart are very rare indeed, if they exist at all. Ether, like chloroform, first stimulates the heart, but it does not, like the latter, seriously depress it afterwards. The great danger in the administration of ether is from the arrest of respiration. His own experiments and observations in the use of chloroform and ether, would lead him to use especial caution in case of the former, to see that there has been no indication of weakened heart's action, as indicated by faintings and dyspepsia; to use especial caution with those addicted to the use of alcohol, and to be particularly careful to give thorough dilution of the vapor till the excitement stage be passed. Finally, to carefully watch the later stages of the anæsthesia, and to suspend the administration at the first indication of any excessive dilation of the pupil, of stentorous breathing, or of relaxation of the muscles of mastication, as these are governed by nerves which arise from the medulla, and indicate danger to the sympathetic system. In ether narcosis, instead of watching so carefully the action of the heart and the pulse, he paid particular attention to the respiration, and at the very first appearance of bronchial rales he suspended the administration. In case of the persistence of unpleasant symptoms from chloroform, the utmost exertion should be put forth to stimulate the heart to action, such as the administration of artificial stimulants, electric currents and nervous shocks. If the danger occurs in the administration of ether, pay little attention to the heart or pulse, but commence artificial respiration, and persist in it. The paper was discussed by Dr. Werner, Professor Atkinson and Dr. Shumway, of Plymouth, after which the Association adjourned for the trip down the harbor.

---

THIRD DAY—EVENING SESSION.

The body was called to order at 8½ o'clock. The minutes were read and approved.

An invitation to attend the Art Museum was extended to the members of the Association by Mr. C. G. Loring.

A committee was appointed to draft resolutions on the death of Dr. H. L. Sage, of Bridgeport, Conn. Dr. Jos. Smith, Dr. W. H. Jones and Dr. James McManus were appointed. Dr. Atkinson called the attention of the Association to the depleted condition of the treasury, and said that there was barely enough to pay for publishing the records of the transactions. He moved that the Secretary's and Stenographer's reports be given to the journals upon the best terms obtainable by the Publication Committee, who should have charge of the business. Dr. Shepard stated that the finances were in a better condition than for several years. Enough money had been taken this year to pay the expenses, and leave about $400 for the publication of the transactions. Secretary Cushing said that Dr. Atkinson's motion was contrary to the constitutional amendment which had been adopted on the first day of the session. Dr. Taft offered a resolution that a committee of five be appointed to consider the feasibility of an international dental congress. Dr. Taft represented such a congress as having possibilities of great benefit to the profession both here and abroad. The resolution was adopted unanimously. Dr. Atkinson's motion having been reduced to writing, was presented. Dr. Taft thought the proceedings could be published without expense to the Association. Mr. Ransom, of Toledo, will publish the proceedings, with advertisements and copies for his own sale, and furnish the members with a printed copy each, without any expense to the Association. Dr. Taft moved that Mr. Ransom's proposition be accepted as a substitute for Dr. Atkinson's motion. Dr. Crouse opposed the substitute, preferring to leave the matter in charge of the Publication Committee. The whole subject was tabled till morning, in order that Mr. Ransom's proposition might be submitted in writing.

Dr. Thomas offered a resolution that the vote of paying one hundred dollars salary each to the Secretary and Treasurer be rescinded from and after this year. Dr. Thomas stated that this meeting was the largest for a dozen years; that the finances were not in good condition, and that the motion contemplated a reduction of the five-dollar entrance fee. Dr. Crouse moved to lay the resolution on the table. It was lost—yeas, 34; nays, 35. Dr. Taft spoke for the resolution, saying that men were away who ought to be in the Association, who objected to coming so long as the officers were salaried when the finances were in bad condition. First-class men were away on account of this feeling. Dr. Atkinson did not think the Association lost much by the absence of such men. If a man was not willing to pay five dollars for the privilege of belonging to the Association, he was in a condition of hopeless insanity. President Shepard, having called a member to the chair, said he believed that every meeting could be made as successful as this by organized work for it, as had been done for this. Causes have been in operation for the past years to make the meetings small, which will not be present in the future. He hoped the resolution would be voted down by a majority so unanimous that it would never be introduced again. Dr. Crouse believed in paying the Secretary and Treasurer, for their work, especially the former's, was very onerous. Dr. Taft defended the resolution. Dr. Thomas said the Secretary's work was worth more than $100, but he made the motion thinking young men would accept the offices for honor alone, and also thinking that the money left over would be enough to pay the officers as much or more than before. The motion was then put and lost. A division of the house showed 19 yeas and 41 nays.

Miscellaneous business having been ended, the discussion of Dr. Barrett's paper was resumed, the doctor taking the floor himself to express his thanks to Professor Mason, the Gamaliel at whose feet he has sat, for the great benefit he has received from him. The question came up whether nitrous oxide would oxidize the blood, Dr. Barrett holding that many dentists believed that it would, though he believed that it would not. Dr. Brophy said it would prevent oxidization of the blood. Dr. Wilson said he thought many dentists in the country did not know the composition of the atmosphere. Dr. Buckingham spoke upon the action of the several anæsthetics upon the blood, saying that the profession has not yet arrived at sufficient knowledge of the chemical action of these substances upon the blood. Dr. Werner said that no one has ever analyzed the exhalations of persons who have taken nitrous oxide, and it is a question whether that gas in pure form is ever administered, because it is so very difficult to keep the gas pure. Motion was made by Dr. Merriam that Dr. Barrett be allowed five minutes in which to close the discussion. Dr. G. F. Waters moved an amendment, that he have all the time he wanted. The motion was carried. Dr. Barrett then declared the discussion closed, amid loud applause, and moved to proceed to the next order of business. Carried. Dr. Taft moved a suspension of the regular order of business to take up a volunteer essay by Dr. Marvin. The subject was "The Unwritten Code of Dental Ethics." He emphasized the duty of sustaining the dignity of the profession; of abstaining from indecently bold advertising of one's abilities (though the duty of discharging his obligations to the public and the profession was asserted) ; of refraining from superciliousness toward the younger members of the profession; of not consuming wastefully the valuable time of men by worthless talk, in the association or elsewhere; of being just to both the profession and to the public. Dr. Marvin enjoined careful scientific attainments, thoughtful language, a cleanly person and gentlemanly deportment as necessary to the honor of the profession. He was warmly applauded as he closed.

Section 3, on Dental Literature and Nomenclature, Dr. W. H. Atkinson, Chairman, was called, and he read a paper on the universal language.

Adjourned.

---

FOURTH DAY—MORNING SESSION.

The Association met and was called to order at 9:45 o'clock. The minutes were read and approved.

Dr. Atkinson's resolution of Thursday evening was adopted without discussion. It provides that the Publication Committee be authorized to confer with publishers to secure the best terms possible in the publication of the transactions, by giving them such portion of the matter as they may wish, as a part or as a full compensation, leaving it discretionary with the Publishing Committee to accept or reject any or all offers.

Dr. J. N. Crouse, of Chicago, presented the report of section 2, on Dental Education. It urged that the Association should fix some standard of preliminary education to be required by dental colleges; that, in addition to the knowledge acquired in the academies, the student should have some office training and a preparatory course of reading before attending lectures. With reference to the dental colleges, it was urged, first, that they should insist upon a more rigid preliminary examination as to the student's character and mental acquirements; second, more care in the examination and graduation of students, which would be secured by selecting the examiners from the best men in the profession. The section was of the unanimous opinion that a higher standard of education would be attained if the schools would require two full courses of lectures before allowing the student to come forward for examination and graduation, and recommended the adoption of the following resolution, namely: "That, in order to secure representation in this Association, dental colleges must, subsequent to October 1, 1881, require all students entering therein to take two full courses of lectures previous to coming forward for examination and graduation, and shall state the same in their next annual announcement." As far as this section has been able to ascertain, the following-named schools had substantially adopted the resolutions passed at the last previous session of the Association, which declared that no dental college should be entitled to representation in the Association that does not require a good English education as a preliminary qualification for its matriculants, and that the colleges should grant no diplomas except to those who attend at least two full courses of lectures, viz: The dental department of the University of Pennsylvania, the dental department of Vanderbilt University, the Boston dental colleges, and the Western Dental College. The dental school of Harvard University and the dental department of Michigan University were already carrying out such restrictions. Such schools as were not responsible, and were a positive hindrance to educational advancement, should be so thoroughly exposed as to make them harmless The question whether dentistry was a specialty of medical science or not had been argued pro and con, and two papers were read on the subject, submitted by Dr. Brophy and Dr. L. C. Ingersoll, of Keokuk. The former argued the necessity of dentists having a thorough medical education and a complete knowledge of dental surgery. Dr. Ingersoll's paper advocated the separation of medicine from operative dentistry.

Dr. Allport advocated improvement in the dental colleges, and a division of branches of dentistry into mechanical and operative, or surgical. Such as intended to practice artificial dentistry should be educated in the dental colleges, and such as were to practice oral surgery should be educated in medical colleges. Until this system was established, people would not be willing to go to two different practitioners for treatment in those two branches. His wonder was not that the standard of the colleges was not higher, but that it is as high as it is. It was important that students in dentistry be taught chemistry, metallurgy and general knowledge of mechanics. Students were required to perform the most delicate operations in mechanical construction, and that without any knowledge of mechanics. He contended strongly that dental surgeons should not be required to make artificial teeth.

Dr Morgan held that the proposition to separate mechanical from surgical dentistry was practically impossible.

Dr. Buckingham agreed with the last speaker, and observed that the colleges could only teach principles. Dr. Stockton thought it was a fact that the profession owed more to the dental colleges than to any other means for fitting students to be dentists. The education of students who entered the profession through the examining boards was not sufficient to make them fit dentists. He thought they should, as far as they could, put their foot down upon these State examining boards. He knew of young men now in the profession who had been admitted by the boards, and who were unable to spell and write correctly. Dr. Moore, of Columbia, S. C., supported the dental colleges. Dr. Templeton, of Pittsburg, held that the reason why so many of the students were unfit to become dentists, was because they came from dentists' offices, where, after serving a short time as "office boys," they had taken a notion they wanted to be dentists, and so went for examination, and were admitted.

Dr. Barrett asked that they should be honest. They were not medical men, and were not acknowledged as such by physicians. There was no way that a student could become a member of the medical profession except through a medical college; that was the law. Because there is the practice of surgery and the use of some medicines in dentistry did not make a dentist a medical man, any more than a shoemaker was a medical man; for a shoemaker, if he be an intelligent member of his noble profession, understands the anatomy of the human foot. He gave way to no man in the honor he accorded to the dental colleges; but they were not by any means what they ought or might be. He believed that the day would come when dentistry would be a branch of university education.

Dr. Wetherbee related the statement of Oliver Wendell Holmes and Professor Bigelow, who would not admit that there was any necessity for students of dentistry to have a practical knowledge of anatomy. He advocated the teaching of medicine to dental students. He would give the colleges their due, but wanted them improved.

Formation of sections was next in order, but so many of the Association had departed that the section ot each member can not be given. Those who remained arranged themselves as follows: Section 1: Artificial Dentistry, Metallurgy and Chemistry—Drs. Stockton, Grant, Carroll, Baker, Fundenburg, Eben Flagg. Section 2: Dental Education—Drs. Shepard, Essig, Crouse, Pierce, Fillebrown, Chandler, Cook, H. A. Smith, McDougall, Mills, Templeton, Brophy, Talbot, Moore. Section 3: Dental Literature and Nomenclature—Drs. Atkinson and Taft Section 4: Operative Dentistry--Drs. Webb, Osgood, Wetherbee, Page, Darby, Watkins, McCampbell, McKellops, Mariner, George L. Field, Colgrove, Leavitt, Priest, Friedrichs, Whitten, Merriam. Section 5: Anatomy, Physiology, Histology, Microscopy and Etiology—Drs. Dean, Barrett, Werner, Niles, Waters, Andrews, Stockwell. Section 6: Pathology, Therapeutics and Materia Medica--Drs. Odell, Morrison, Dudley, Harlan. Later in the day the sections organized as follows: Section 1—Dr. Stockton, Chairman; Dr. Carroll, Secretary. Section 2—Dr. Fillebrown, Chairman. Section 3—Dr. Atkinson, Chairman Section 4--Dr. Webb, Chairman; Dr. Darby, Secretary. Section 5—Dr. Barrett. Chairman. Section 6—Dr. Odell, Chairman; Dr. Harlan, Secretary.

Dr. C. S. Stockton, Chairman of Section 1. on Artificial Dentistry, Metallurgy and Chemistry, reported for the section. He said there was a growing interest in artificial dentistry. While the older methods of work had undergone no great change during the past year, improvement was reported in the working of celluloid that rendered this base more certain in its results. The improvement was the perfection of the new mode celluloid machine, invented by Dr. Campbell, of New York, by which the celluloid undergoes a process of curing while the plate is being pressed, the material thereby being made much harder, and less liable to discolor or warp while being worn. In the manufacture of teeth there was a decided improvement in the forms of the bicuspids and molars, some of the manufacturers having the courage to attempt the introduction of the natural form of those teeth, though the best of those attempts left much to be desired. As many advanced in years required the services of the profession in this department, it was suggested that teeth be manufactured that will represent the effect of wear upon the cutting and grinding surfaces. Attention was called to the new metallic base--" Reese gold alloy" —which promised much in the way of accuracy of fit, and a better health of the mouth.

Dr. Flagg, of New York, followed with a paper, in which he urged the study and practice of dentistry as an art, and offered some instructive suggestions. He said that if students would commence the study of their art by carefully selecting models from plaster impressions of well-shaped mouths, the education received from studying them would never permit the acceptance of the chinaware, for which, he was sorry to say, a demand had been established at the dental depots. In reference to the use of rubber he claimed that, through the process by which rubber is worked, it must produce a detrimental effect upon the advancement of anything artistic. He deplored the neglect of this subject, which neglect, he held, had degraded the profession. There was no discussion on this subject, and the Association proceeded to the transaction of business.

The selection of the place for the next annual meeting was now declared to be in order. Several nominations were made. Upon a ballot being had, New York was chosen.

The election of officers, by ballot, for the ensuing year resulted as follows: President, Dr. C. N. Peirce, of Philadelphia; First Vice-President, Dr. W. C. Barrett, of Buffalo; Second Vice-President, Dr. George J. Friedrichs, of New Orleans; Recording Secretary, Dr. George H. Cushing, of Chicago, Corresponding Secretary, Dr. A. M. Dudley, of Salem; Treasurer, Dr. William H. Goddard, of Louisville, Ky.—re-elected for the fourteenth time; Executive Committee, Drs. S. G. Perry, of New York; W. H. Morgan, of Nashville, and T. T. Moore, of Columbia, S. C.

The Association passed votes of thanks "to the local committee for its very admirable arrangements, which have so largely contributed to our comfort and enjoyment during the session of this Association ; to the reporters of the public press for their excellent reports; to the proprietors of Hotel Brunswick for their liberal arrangements and superior accommodations, and to the authorities of the Massachusetts Institute of Technology for their very generous gift of the use of their building for our uses, whereby we have enjoyed unusually good accommodations." The Association also desired "to gratefully express their appreciation of the courtesies of the city of Boston, as extended to them in the sail through the harbor, and to the Mayor for his graceful attentions upon that occasion." A vote of thanks was passed to President Shepard.

Drs. Taft, Cushing, Brownson, McKellops, Shepard and McManus were appointed as the committee to consider the feasibility of an international dental congress. The committees appointed to draft resolutions on the death of Dr. H. L. Sage and Dr. Samuel Stockton White submitted their reports, and the resolutions were adopted. Dr. Morgan addressed the Association, and paid warm tribute to the memory of Dr. White, saying that he had done more than any one else in the profession to make the improvements of the last thirty-five years. By a rising vote, the resolution of respect to Dr. White was unanimously adopted. Votes of thanks were then passed to the officers of the Association. Drs. Atkinson and Taft were appointed a committee to conduct President-elect Peirce to the chair. Dr. Shepard made a brief speech on retiring, and Dr. Peirce a brief one on assuming the chair. The President appointed Drs. Perry, Kingsley and Mills the local Committee of Arrangements, and Drs. Darby and Dean the Publishing Committee.

The minutes of the meeting were read, and the Association adjourned at 2:30, to meet in New York on the first Tuesday of August, 1881.

